# Holographic Duality and the Physics of Consciousness

**DOI:** 10.3389/fnsys.2022.685699

**Published:** 2022-03-15

**Authors:** Uziel Awret

**Affiliations:** Inspire Institute, Alexandria, VA, United States

**Keywords:** holography, duality, coding, meta-problem, physical theory of consciousness, strange metals, dual-aspect

## Abstract

This paper introduces a novel dual-aspect theory of consciousness that is based on the principle of holographic-duality in modern physics and explores the prospects of making philosophically significant empirical discoveries about the physical correlates of consciousness. The theory is motivated by an approach that identifies certain anti-physicalist problem intuitions associated with representational content and spatial location and attempts to provide these with a consciousness-independent explanation, while suspending questions about the hard problem of consciousness and the more problematic “phenomenal character”. Providing such topic neutral explanations is “hard” enough to make a philosophical difference and yet “easy” enough to be approached scientifically. I will argue that abstract algorithms are not enough to solve this problem and that a more radical “computation” that is inspired by physics and that can be realized in “strange metals” may be needed. While speculative, this approach has the potential to both establish necessary connections between structural aspects of conscious mental states and the physical substrate “generating” them and explain why this representational content is “nowhere to be found”. I will end with a reconsideration of the conceivability of zombies.

*But if there is a kind of inherent necessary connection between being the kind of cognitive system we are and possession of the requisite experience, then the problem seems much less serious. What kind of metaphysics this would involve is not clear. The idea would be that there is something in the nature of intentional “scripts” of the kind we instantiate that necessitates an experiential reflection of it — a “script” that is necessarily produced as a movie. In a way, this might be a form of “panprotopsychism” as applied to certain informational states.*

Levine (2019)

## Prolog

We seem to have good reasons to believe that consciousness is physical and good reasons to believe that it is not. This paradoxical situation is referred to by Stoljar (2009) as our “Epistemic situation” (or predicament) and following Post-Newtonians like Priestly who claimed that we don’t know enough about the physical to conclude that consciousness is not physical, Stoljar solution to this epistemic dilemma is to separate the consciousness problem in two, a scientific empirical one (Stoljar, 2006), and a philosophical one aiming to explain our anti-physicalist intuitions. Explaining away such problem intuitions is crucial to Non-Reductive Physicalism, Eliminativism and Strong Illusionism (Frankish) (“The Meta-Problem Challenge”) and also to the conceivability of zombies. Such explanations can be psychological, computational, structural, neurological or cultural, one satisfying class of explanations appeals to the “riches of physics” and embrace a version of Stoljar’s (2006) Ignorance Hypothesis (IH) holding that we are ignorant of physical facts relevant to consciousness whose knowledge would explain away our anti-physicalist intuitions.

The IH comes in different versions that depend on how broad we take the “physical” to be. The “physics” may refer to our standard physics or to a non-standard physics that includes intrinsic or protophenomenal properties. In this paper, unless I say otherwise, I will embrace a first order approximation of the IH that takes the “physical” to be standard. Lets call the physical facts that the IH appeals to—S-facts (after Stoljar). The discovery of such facts is important in its own right because there are good reasons to believe that providing a physical explanation of our anti-physicalist intuitions will constrain solutions to the hard problem (Stoljar’s Epistemic View does not search for possible S-facts but only claims that the IH is the best default explanation of our epistemic situation).

S-facts are not easy to imagine but easier to imagine than the facts needed for a physical explanation of consciousness. To see why S-facts are hard to conceive of consider Papineau’s (2007) critique of the IH:

*Stoljar is here placing strong demands on the content of our ignorance. It must be such that, if only we knew the relevant non-experiential facts, this would render zombies inconceivable. However, it is not clear that any non-experiential facts could play this role. By their nature, non-experiential facts would seem to be third-personal, objective, and non-perspectival, while experiential facts are first-personal, subjective, and perspectival. It is hard to see how knowledge of the former could automatically render the absence of the latter inconceivable.*

It is therefore quite possible that no physical facts can be both strange enough to explain our problem intuitions and “standard-physical” enough to count as standard.

The Strange Metal theory of consciousness (section “Strange Metals”) is an attempt to provide a physical explanation to some of our essential anti-physicalist intuitions and especially, an attempt to conceive of possible empirical findings about the neural correlates of consciousness (NCC) that can make a “philosophical difference” (section “NCC Correlations and Empirical Equivalence”) I will argue that unlike classical physics and QM, which lack the resources to provide S-facts, Quantum Gravity (especially with its unexpected connections to condensed matter theory) does harbor such resources (section “Physics with Resources to Explain the SMP and ∼P Problem Intuitions”).

When it comes to formalizing consciousness by system theorists and mathematicians one can adopt top-down approaches inspired by symmetry (Kleiner, 2020) or by fundamental postulates (Tononi and Koch, 2015). Here, using a physics inspired approach, I begin by searching for a physics that can explain some strategically chosen anti-physicalist intuitions (section “SMP and ∼P Problem Intuitions”) only to discover a highly abstract physics in which information is more fundamental than spacetime and “matter” and “identity” prove subtler that we realized (section “Physics with Resources to Explain the SMP and ∼P Problem Intuitions”). The mathematical structure that is exposed by this appeal to the physics of the meta-problem is relevant to attempting a mathematical formulation of consciousness that expands what we mean by physical system such as to endow those with rich private “inner-world” that is theoretically inaccessible to other systems.

To appreciate the unexpected relevance of quantum gravity to our anti-physicalist intuitions (4-2) I will consider Susskind’s (2017) radical ontological interpretation of the “Anti-de Sitter Space - Conformal Field Theory (AdS-CFT)” correspondence (4-5). In Feynman’s spirit of – “Don’t ask yourself whether it is *too strange* but whether it is *strange enough”* -I argue that Susskind’s interpretation is both physically “standard enough” and “strange enough” to explain relevant anti-physicalist intuitions.

For Susskind entanglement in d dimensions is equivalent to a wormhole in d+1 dimensions, one cannot exist without the other. What is important for our purposes is his thought experiment that includes a spherical shell described by a two dimensional conformal QFT (quantum field theory). Imagine a large quantum computer implementing complex intractable computation (4-6). According to the AdS-CFT correspondence the spherical shell is dual to a three dimensional inner “bulk” AdS space (4-1). Susskind notes that this dual bulk space is different than the laboratory space. The bulk space implements what is essentially the same computation as the quantum shell but in a completely different way. While there is an information theoretic sense in which the dual spaces are identical, physically they could not be more different. Each element in one space maps unto a unique element in the dual space (bijection) and while an element may be fundamental, or local, in one space, its dual may be composite (relativity of fundamentals) or non-local (4-1). For our purposes what is important is that the only way to access the rich representation in the bulk is to have a “technician” uploaded unto the surface quantum computer. Susskind goes on to speculate that such a technician, once entering the bulk, could find ways to communicate with the lab technicians. However, the bulk space which is real, having a novel relationship to the lab spacetime, can also provide us with a physical subjective space harboring rich representations that evolve in parallel to the on goings of the quantum shell computer.

The question I will ask is whether consciousness can inhabit such “bulk” AdS spaces and whether such “physics” can explain why it is so hard to believe that consciousness is identical to anything in the brain [and is nowhere to be found in Leibniz Mill (section “SMP and ∼P Problem Intuitions”) Such physics may also provide necessary connections between the more structural aspects of our phenomenology and corresponding structural aspects of the brain. One way or another a putative “physics of consciousness” will have to explain the relationship between the “space of phenomenology” and the “phenomenology of space” (section “Physics with Resources to Explain the SMP and ∼P Problem Intuitions”) or what Chalmers (2020) describes as reconciling the *scientific* and *manifest* images of space] and here AdS-CFT has one more connection to our phenomenology that should interest system-theorists, It enables us to view rusted pieces of copper oxide as computing devices that “convert” hard, intractable, quantum, information inaccessible by perturbative methods, into easy, classical, *geometrical*, phenomenological information accessible to perturbative methods (4-4). If we were to discover that our minimal PCC is describable by such CFT it would be hard to reject the possibility that the centrality of geometry to our spatiotemporal phenomenology results from such dual bulk AdS spaces. System theoretic approaches to consciousness need to take a stand on the privacy or “radical interiority” of consciousness. Some like Tononi (2008) claim that any system with non-zero Phi has unique (extra-theoretical, non-falsifiable) access to itself not available to any other system, while some like Clark (2019) use predictive coding to attribute our anti-physicalist intuitions to the unavailability of interoception to experience.

Another way in which I hope this paper will be relevant to system theorists is as an example of a meta-theoretic strategy conjoining putative solutions to the hard problem with topic neutral explanations of our anti-physicalist intuitions (section “The Meta-Problem of Consciousness”). As we will see system theorists interested in the hard problem may want to first construct (honest) system-theoretic models that generate anti-physicalist intuitions similar to ours and only then use this construction to constrain the hard problem (section “The Meta-Problem Challenge”). While the first step is empirical the second step is more philosophical and embraces Chalmers claim that the Meta problem and hard problem of consciousness are “almost independent” but co-constraining. As a matter of fact a fruitful way to think of a solution to the Meta problem of consciousness is that of showing that a “smart enough” honest embedded AI is likely to generate conscious and problem reports similar to ours.

## Introduction

The biggest obstacle standing in the way of a “mathematical formalization of experience” is probably related to David Chalmers’ “Structure and Dynamics Argument” (Chalmers, 2002; Alter, 2015) and similar to:

a) Mathematics is structural.
b) Structure can only yield more structure.
c) Consciousness is not structural.
d) Conclusion: Mathematics cannot describe consciousness.

One way to reject this conclusion is to reject the first premise by arguing that mathematics may indeed include non-structural entities too. Another way out is to reject the third premise and search for relevant structural aspects of conscious experience. I will follow the second path and concentrate on three points of contact between mathematics, modeling and experience:

1) Distinguishing two major properties of consciousness—The non-structural “phenomenal character” common to all conscious states and the more structural “representational content” that differentiates conscious states.
2) Distinguishing between the “hard problem of consciousness” and the more empirical “meta-problem of consciousness.”
3) Searching for physics that is rich enough to solve the meta-problem.

Sec. “Ineffable and Manageable Anti-physicalist Intuitions” explores the first point of contact. “SMP and ∼P Problem Intuitions” presents what I term the “Structural Mismatch Problem” (SMP) and the “∼P Problem.” “Magic and Necessity” presents a thought experiment correlating holography and necessity, while “Coding and Necessity” asks whether “deep-learning-based” coding strategies can solve SMP. Sec “NCC Correlations and Empirical Equivalence” explores causation and correlation in the NCC.

Sec. “Empirical Challenges” explores the second point of contact. “The Meta-Problem of Consciousness” introduces the meta-problem of consciousness, “The Meta-Problem Challenge” considers more empirical approaches to the meta-problem and gauges its philosophical impact. “The Meta-Problem Challenge” finally argues that the meta-problem pressures Russellian Monism.

Sec. “Holographic Duality and Strange Metals” combines the first two sections to search for physics that solve the SMP and the ∼P Problem, concluding that the phenomenon of “Holographic Duality” in modern physics has the resources to explain these intuitions. I shall introduce some concepts in and around the modern physics’ treatment of duality in order to promote, in the final discussion, a “holographic theory of consciousness.” “Duality” introduces the principle of duality, “Physics with Resources to Explain the SMP and ∼P Problem Intuitions” argues that “Holographic Duality” is suitable to the presented problem. “Strange Metals” introduces the concept of “Strange Metals” and the “AdS-CMT correspondence,” “AdS-CFT Correspondence as Computation” introduces the AdS–CFT correspondence as pushing the envelope of computation, “Einstein-Rosen bridge (ER) = einstein podolsky and rosen (EPR)” portrays the “ER = EPR interpretation of entanglement” and a more radical version of duality, and “Holographic Duality and Quantum Error Correction Codes” describes the connection between quantum error-correcting codes and the constitution of spacetime.

I will end with a discussion in which I will ask whether the suggested “holographic” theory of consciousness can handle zombies.

## Ineffable and Manageable Anti-Physicalist Intuitions

### Structural Mismatch Problem and ∼P Problem Intuitions

Consciousness is roughly attributed two kinds of properties: *phenomenal character*, common to all conscious states, and *representational content*, specifying the difference between such states.

The phenomenal character consists of properties common to all conscious states, including the feeling that *there is something it is like to be conscious*, or that consciousness is given to a self, or to itself, or that it is directed, or transparent to introspection, or that it is self-affirming. The representational content refers to the difference between such states.^1^ In section “Coding and Necessity” I will present recent work suggesting brain-bound explanations of some of the structural aspects of our phenomenology.

The question ‘‘What is it about the way phenomenal experience *is* that is made necessary by the way the brain *is*’’?^2^ can be broken into two:

a) What is it about the way phenomenal character *is* that is made necessary by the way the brain *is*?
b) What is it about the way representational content *is* that is made necessary by the way the brain *is*?

The first point of contact between mathematics and consciousness that I want to consider is question (b) – because, unlike (a), it relates two domains with structural properties. Hence, in this section I evaluate the prospects of establishing necessary connections between the structural aspects of representational content and the structure of the brain states that generate (or correlate with) it.

Already in 1714, in Sec. 17 of his *Monadology*, Leibniz (1960) argues that perception cannot be given a “mechanical” explanation:

One is obliged to admit that perception and what depends upon it is inexplicable on mechanical principles, that is, by figures and motions. In imagining that there is a machine whose construction would enable it to think, to sense, and to have perception, one could conceive it enlarged while retaining the same proportions, so that one could enter into it, just like into a windmill. Supposing this, one should, when visiting within it, find only parts pushing one another, and never anything by which to explain a perception. Thus it is in the simple substance, and not in the composite or in the machine, that one must look for perception.

There are two things that you will not find in the brain. First, consciousness itself, which is only given to the “owner” of the brain (i.e., the phenomenal character of consciousness). Second, and just as importantly for our purpose, representational content; if the owner of the brain is conscious of three blue goats, anyone in the windmill shall be hard pressed to find a blurry imprint of three goats or anything resembling that. If the image is physical how can it be identical to something in the brain? I will concentrate on two “problem intuitions” that are related to Leibniz’s Mill. The first is the “The Structural Mismatch Problem” (SMP), following Chalmers’ (2016) use of the term in the context of the “combination problem” of constitutive Russellian Monism, yet applying it more generally. The SMP is the intuition that the structure of the representational content of phenomenal states lacks any necessary connections to the brain structure that generate them. This phenomenal field that can harbor a huge number of “qualia” combinations can be considered as a system of differences endowed with structure and information laden. Even eliminative materialists that view qualia as illusionary would agree that it is at least a richly structured illusion utilized by the brain for executive function. At the same time, the physical or neural substrate generating such “phenomenal fields” has its own structure that is completely different from the phenomenal structure. All major theories of mind suffer from an inability to establish or even conceive of necessary connections between these two structural domains, or sets of differences, despite their exquisite correlations.

The second central anti-physicalist problem intuition is what I call the “∼P Problem,” or “not Physical” problem intuition, to borrow from Levine (2019)—*why mind-body identities provoke cognitive dissonance in a way standard theoretical identities don’t.*

The ∼P problem intuition is also related to the “other minds problem”: how can complex physical systems generate complex physical self-representations accessible to those systems but completely concealed from “without”? As we will see, the same physics that I argue to be rich enough to solve the SMP is also rich enough to solve the ∼P Problem intuition, providing the only physical account that I know of such “private” spaces.

Both are important anti-physicalist problem intuitions and, as we will see, constructing consciousness-independent explanations to these problem intuitions is an important part of a multidisciplinary meta-problem research program concerning philosophers, system theorists and computational neuroscientists.

One way of explaining these problem intuitions is explaining why the necessary connections between mentality and the brain are hidden. Let us consider the next “holographic metaphor.”

### Magic and Necessity

Uri Geller hears about a planet that is pretty advanced but with inhabitants that have not yet discovered holography and the Gabor transform (a linear transformation relating a 2 dimensional hologram to its associated 3 dimensional holograph). He loads his spaceship with holograms and a laser and takes off. As soon as he arrives, he announces that he will perform an act of real magic. The mostly naturalist inhabitants, embracing continuity and rejecting radical emergence, gather around with curiosity. Geller pulls out a thin glass plate (hologram) and shines a laser through it producing a 3D holographic image. The inhabitants are surprised but are sure that the holographic image emerges from the holographic plate; they tell Geller that, however, surprising this might be, this phenomenon must have a logical, physical explanation. Geller retorts that at the very least they have a serious structural mismatch problem, because the patterns of the face carved on the surface of the hologram plate have nothing to do with the holographic image and do not seem to necessitate it in any way. He proceeds to challenge them to conceive of any possible necessary connection between the two. He also shatters a hologram into pieces and shows them that shining the laser through the fragments produces low resolution holographs of the whole image, and then challenges again the locals to explain it [especially the large local panpsychist community who believe that their biggest problem is explaining how elementary “little subjects” combine into a large subject (Coleman, 2014)].

The inhabitants happen to have powerful computers with decent big data and deep learning capabilities, and confiscate the holograms to explore the correlations between the holograms and the holographs. After a couple of months, they are able to predict the images generated by holograms that they have not analyzed before (They kept a couple for that purpose).

The inhabitants of the planet tell Geller that they can generate these images by using their computers and that this is not real magic, to which he responds that the production of mere correlations does not provide necessary metaphysical connections between the plates and the holograms and that they still have a serious structural mismatch problem. The inhabitants go back to the lab and use information compression algorithms to search for the most efficient way of constructing holographic images from previously unseen holograms, with the constraint that the fragments produce lower resolution holographs of the whole image. After another month, they discover the Gabor transformation in which the hologram necessitates the holograph, learn about phase-front reconstruction and fine Geller in Bitcoin.

### Coding and Necessity

Here we can ask whether something similar can be done with neural decoding, in which deep learning and big data approaches can be used to determine what face a primate is looking at among thousands just by “observing its brain.” The last 20 years have witnessed a steady improvement in our ability to decode the subjective contents of the human brain, from *Distributed and Overlapping Representations of Faces and Objects in Ventral Temporal Cortex* (Haxby et al., 2001) to *Identifying Natural Images from Human Brain Activity* (Kay et al., 2008), to *Reading the Mind’s Eye: Decoding Category Information during Mental Imagery* (Reddy et al., 2010), to *Neural Portraits of Perception: Reconstructing Face Images from Evoked Brain Activity* (Cowen et al., 2014), to more recent work based on both single neuron measurements, e.g., *The Code for Facial Identity in the Primate Brain* (Chang and Tsao, 2017), and more global fMRI methods, *Reconstructing Ffaces from fMRI Patterns Using Deep Generative Neural Networks* (VanRullen and Reddy, 2019). These new decoding capabilities fall short of solving the SMP but are not just mere brute correlations. At their best, such deep-learning-based decoding can identify the optimal dimensionality of such spaces. In Chang and Tsao’s (2017) code the “computer” identified a 50-dimensional face space, and facial images are thus expressed as points in a 50-dimensional space. What was surprising about this work is that identifying the dimensionality of the “face space” and the relevant neural correlates enabled recording from a few hundred neurons to accurately decode a large number of faces. This may be attributed to the conjecture that deep learning and evolution settle on the same number of dimensions. It also seems that similar linear code strategies are ubiquitous to biological pattern recognition. While such codes do not help solve the meta-problem of consciousness or the SMP they do seem to support the claim that representational content possesses a structure that lends itself to scientific investigation.

The decoding of visual images like faces has the advantage that it yields a “metric” in which the degree of difference between images is commensurate with the difference in neural firing patterns that can be expressed as a distance between two points in some abstract space. When faces are similar, the activation patterns are similar and their distance is short. One way of constructing such “neural manifolds” that admit a metric and a distance formula is using *statistical geometry* (Roy and Kafatos, 2003).

The difference between faces is easier to formulate than the difference between scents. However, that has not stopped olfaction researchers from constructing a metric for the “distance” between different scents on an olfactory scale. Olfaction researchers deal with two transformations, from the physical space of odor molecules to the space of neural activity and from that to the space of odor perception. To formulate such transforms we need to first construct metrics for these three different spaces. In *Measuring Smells* (Haddad et al., 2008), the authors take two different approaches to constructing a metric for structural chemistry in which the ‘‘Dragon’’ software^3^ is used to obtain 1664 molecular descriptors for more than 1500 odorants. Each odorant is described by a vector in a 1664-dimensional space and subjected to a principal component analysis as a well-established method for dimension-reduction. As for this study, each odorant in the physical space can be described in two ways, one where the distance between odorants is determined by their principal component score (PC1) and one by taking the full vectors and measuring the “geometric distance” between them (summing the squares of the differences between the components and taking their square root). The application of principal component analysis to all three sets of data showed that the key axis (PC1) was correlated across all three domains, providing a one-dimensional metric based on pleasantness that could also be extended to other species. The second multi-dimensional metric construction was successful in predicting neural activity and showing that the smaller the distance between odorants in the physic/chemical space, the more similar the neural responses. This metric too seems to be applicable to different species, suggesting that aspects of the structure of olfactory space are conserved across species based on the reliance on similar environmental regularities.

Olfactory decoding is not good at explaining why a rose smells the way it does, more importantly, nor does it help solve the SMP and ∼P Problem intuitions. I presented these studies in the olfactory field as these findings are analogous to the visual field, which is more relevant to the purpose of this paper. This is because visual perception is more geometric than olfaction, and as we will see in Sec. “Holographic Duality and Strange Metals” where geometric structure emerges from “holographic computation.”

It seems as though such brute computerized decoding strategies can at most tighten the correlations between phenomenal states and the neural (physical) processes that generate them; yet, can they reveal, a deeper overarching small set of psychophysical laws? Perhaps because information compression is evolutionary advantageous and because Deep Learning neural nets and their layered architecture are good at optimizing information compression. Here is Joseph Levine (2019) on the “fine-tuned structure of experience”:

*“Among the materialist arguments that I find most compelling is the appeal to the myriad ways in which what I will call the ‘finetuned structure’ of our conscious experience can be explained to a very large extent by the functional profile of the underlying physical mechanisms. For instance, take color experience. Leaving aside the explanatory gap that attends there being any experience at all, or one’s color experience having the particular qualities that it does, there is clearly a lot about the structure of that experience that is explicable by appeal to underlying physical-functional mechanisms. …It’s reasonable, then, to suppose that whatever psychophysical emergent laws there are possess a unified structure that makes sense of this relation between physical-functional architecture and the fine-tuned structure of experience.”*

Can deep-learning and big data provide us with that “unified structure” and proper psychophysical “laws of nature.” There is a sense in which improvements in such methods can at best make strong emergence harder to accept. Even cases in which deep learning can discover invariant properties in the data, like optimal dimensionality that both facilitates information processing and is shown to be harnessed by the brain, are “environmentally opportunistic”; thus, they lack the resources to reveal a mathematical transformation that explains away the SMP in the way that the Gabor transformation explains away the mystery of holography. So, while brute decoding lacks the resources to solve both the hard problem and the meta-problem of consciousness it does show that representational content can be accessed scientifically and modeled.

We are still nowhere close from an answer to the question that framed this section: What is it about the way the structure of our phenomenal states *is* that is necessitated by the structure of the physical substrate generating it? Nor are we closer to explaining away the SMP and ∼P Problem intuitions.

The classical hologram-holograph relationship is based on phase front reconstruction and can perhaps be related to neuronal processing but still relates two physical domains. It builds as best an analogy with the relation between phenomenal states and their neural correlates. This begs the question, if the “Technicolor” phenomenal domain is indeed physical, we need to understand why it is so hard to accept as physical, and hopefully physics is rich enough to explain that. To try and answer this question I will argue that we need a more radical computation instantiated in a more radical system by more radical physics. In Sec. “Holographic Duality and Strange Metals” I will still appeal to a holographic correspondence of sorts and yet a radically different one.

### Neural Correlates of Consciousness Correlations and Empirical Equivalence

The claim that frames this whole discussion is that there are physical processes whose instantiation by the NCC could make a “philosophical difference” by explaining relevant anti-physicalist intuitions. The physics that I will rely on to do that allows for very strange correlations in which two complex entities A and B are highly correlated without A causing or constituting B and vice versa. Neither can such correlations be explained by appeal to third party explanation. To see why such physics can make a “philosophical difference” let’s consider Kriegel’s (2020) “Beyond the Neural Correlates of Consciousness” in which he argues that explaining the correlation between A and B is exhausted by:

1) A causes B or B causes A.
2) A constitutes B or B constitutes A.
3) A and B are caused by a third party.
4) A and B are constituted by a third party.

I will argue that explaining the correlations involved in “A is Dual to B” (loosely related to the correlations typical to QM entanglement) does not fit into any of these categories. Kriegel goes on to argue that:

a) When it comes to mind-brain correlations we are not likely to discover empirical facts about the NCC that will favor causal explanations over constitutive ones or vice versa.
b) The six possible explanations of the mind-brain correlations that appear in 1-4 map out our standard theories of mind.
c) It is not likely that we will discover empirical facts about the NCC that will favor one theory of mind over another.

Kriegel’s “empirical equivalence” can be interpreted both pessimistically and optimistically, the pessimist may conclude that we are not likely to discover philosophically relevant empirical NCC facts while the optimist can accept premises a-c but argue that there are explanations of the correlation between A and B, missed by 1-4, that map unto theories different than standard theories of mind. Not only is it possible that we will find empirical facts about the NCC favoring such theories, those among us that search for a philosophically significant physical explanation to the mind-brain correlations seem to gain an important hint; the physics explaining these correlations cannot be based on causation or constitution nor on simple third party explanations. If one accepts 1-4, but refuses to believe that we will not discover philosophically relevant empirical facts about the NCC, such “novel” theories becomes especially attractive. This means that if condensed matter physicists were to discover materials that display such “exotic” correlations it would be worth asking whether similar physics is instantiated by the NCC (including its biomolecules and electron clouds).

Kriegel believes that the theories of mind mapped by 1-4 are empirically equivalent because in the case of NCC correlations it is hard to imagine empirical findings about the NCC that favor constitution over causation (or vice versa). Roughly, the reason for that is that the difference between causation and constitution boils down to establishing the presence, or absence, of either a causal mechanism or time-delay and it is hard to think of an experiment that can establish the existence (or lack) of a causal mechanism or time delay in the case of mind-brain correlations. A similar argument applies to the third party explanations.

There are other reasons to think that the correlations between brain states and their correlated phenomenal states cannot be described by 1-4 and all this suggests that we need to consider bulk physics exhibiting correlations that do not fit comfortably into 1-4. This is precisely what happens with the physics of holographic duality and the AdS-CFT (“necessary correlations”) correspondence that not only transcends causation and constitution but has other philosophical advantages.

Discovering that the NCC harbors physical mechanisms instantiating holographic duality (like in the putative case of the Strange Metals) would provide an example of a philosophically significant empirical finding related to the NCC that is interesting *even* as a thought experiment because it shows that philosophically relevant empirical discoveries about the NCC are possible.

What is important for our purpose is to show that correlations typical of Holographic Duality cannot be described by 1-4 because such duals neither constitute nor cause each other, nor are they constituted or caused by a third party, convincing Vistarini (2017) that such a relation is better described as a Dual Aspect theory.

Radical Duality suggests that the same information is realized in completely different ways and “simultaneously” so…

In Sec “Duality” I will argue that CFT-AdS provides such a connection and constitutes such a metaphysics. To conclude, the “physics of consciousness” should explain correlations that are not explained by Kriegel’s 1-4. In Sec “ER = EPR” we will consider Leonard Susskind’s thought experiment, relating entanglement in QFT to the Einstein-Rosen Bridge connecting black holes as a radical example of such thinking.

## Empirical Challenges

### The Meta-Problem of Consciousness

The second point of contact between physics and consciousness derives from the consideration of the “meta-problem of consciousness”—seeking to provide topic neutral explanation to what we say and know about consciousness—a strategy used by philosophers including Hobbes, Hume Spinoza and also Kant (1781/1999); Place (1956), Armstrong (1968); Dennett (1992), Rey (1996); Carruthers (2017), and lately Kammerer (2018) and Frankish (2019). The meta-problem is situated “in between” the hard and easy problems of consciousness. This *empirical* problem constrains the hard problem while lending itself to mathematical modeling.

We may be witnessing the beginning of a trend in the philosophy of mind in which metaphysical theories of consciousness aiming to solve the hard problem of consciousness (i.e., how matter gives rise to the mind) must also explain “conscious reports” and “problem reports,” or the problem of how consciousness acts back on the matter of the brain to become the source of what we know and report about it. A successful theory of introspection should both secure the foundations of our self-knowledge and explain how consciousness manages to generate reports about itself. In the introduction to their *Introspection and Consciousness*, Smithies and Stoljar (2012) write:

Recent philosophy of mind has been dominated by metaphysical questions about the nature of consciousness and its place in the physical world, while much less attention has been devoted to questions about the epistemic role of consciousness as a source of knowledge and justified belief.

Recent work attempting to conjoin the metaphysics of consciousness and the epistemology of self-knowledge (Stoljar, 2016; Byrne, 2018) has culminated in Chalmers’ (2018) *The Meta-Problem of Consciousness*. According to Chalmers, the meta-problem of consciousness is the problem of why we think that the problem of consciousness is hard or why we think that the explanatory gap associated with consciousness is categorically different from explanatory gaps between different sciences. Chalmers sharpens this more unified approach by concentrating, as a first order approximation, on topic-neutral (independent of consciousness and its cognates) explanations of our *problem intuitions*. We can think of solutions to the meta-problem as accounts explaining why “smart enough” unconscious AI is likely to generate “conscious reports” like “I am conscious!” and problem reports like “I cannot believe that consciousness is physical”. “Problem intuitions” can be viewed as the underlying artificial machine states that cause such reports. A strong illusionist about consciousness like Frankish (2019) refers to those as “quasi-phenomenal,” whereas a strong emergentist like Joseph Levine (2019) refers to them as “intentional scripts,” being indeed directed either at the world or at other internal states of the machine, however, synthetic their origin.

The meta-problem is situated in between the hard and easy problems. It is “easy” because it is independent of consciousness, and yet it is “hard” because it requires explanations on how non-conscious systems can generate conscious reports and problem reports that are indistinguishable from ours. The meta-problem is thus not only the hardest “easy problem” but, as Chalmers (2018) shows, one constrains the possible solutions of the hard problem.

Chalmers’ procedure aims to test the coherence of theories of mind by demanding that metaphysical theories of consciousness clarify their position on the meta-problem The existence of a solution to the meta-problem of consciousness is crucial to both eliminativists like Dennett and to proponents of the conceivability argument like Chalmers (because for a zombie to be conceivable it must generate conscious and problem reports). The meta-problem program is a multidisciplinary attempt to provide topic-neutral explanations to our problem intuitions, drawing from naturalistic, computational and philosophical insights. The problem intuitions giving rise to the hard problem can depend on culture, language and more. Naturalist explanations can appeal to the biological evolution of self-modeling, counter-factual emulation and the modeling of space and time.

The meta-problem also challenges computer scientists. In a recent paper, *Consciousness as Generative Entanglement*, Andy Clark (2019) lists the many successes of predictive processing in modeling perception, action and attention, adding:

But despite their clear promise as accounts of the neurocomputational origins of typical and atypical forms of human experience, they have not yet been leveraged so as to shed light on the so-called hard problem of consciousness…

Clark (2019) suggests that predictive processing is ideally suited to solve the meta-problem by explaining why it is likely to report “puzzling qualia”:

The upshot is that the contents that constitute our qualitative experiences are subtly responsive to our own reactive dispositions and current physiological state in ways that remain hidden from us.

These involve complex cascades of interoceptive and proprioceptive predictions whose inaccessibility to introspection causes us to attribute their origin to something non-existent similar to Armstrong’s ‘‘headless woman illusion,’’ where failing to see the woman’s head is confused with seeing the woman without a head. Here the fact that qualia seem to emanate from nowhere causes them to seem puzzling.^4^

Carl Friston’s (2018) *Am I Self-Conscious?* takes a different route, where the most essential property to conscious self-modeling is “temporal thickness” [also see Chouraqui (2011)]:

*The proposal on offer here is that the self-evidencing has a temporal thickness and depth, which underwrites inferences about the counterfactual consequences of action. This necessarily lends (active) inference a purposeful and self-centered aspect that has the hallmarks of consciousness.*

Friston is an eliminativist trying to provide a topic neutral explanation to the hard problem of consciousness and not to the meta-problem like Clark; however, what is interesting here from a modeling point is that Lisman (2005) and Lisman and Buzsáki (2008) “theta precession inspired” Temporal Coding mechanism seems to provide such temporal thickness that can also be related to Gregory’s “presence” and useful for modeling indexical concepts (“here” and “now”). These are all topic-neutral contributions and it is hard to see why an AI with an architecture inspired by place and grid neurons may suddenly spring to consciousness. Combining advanced versions of self-modeling, temporal modeling and the modeling of counter-factual emulation is part of the meta-problem of consciousness program.

The meta-problem strategy is ideal for modes of analysis that combine *a priori* analytic approaches (like the six types of theories of mind comprising Chalmers’ “A-F taxonomy”) with empirical ones. It is not a naturalistic strategy to reduce our immutable philosophical concepts to scientific reconstructions; rather, the aim is to suspend such philosophical investigations, to first solve the meta-problem empirically, to account for how these appeared in beings like us (Awret, 2019).

### The Meta-Problem Challenge

The meta-problem of consciousness challenges theories of mind with a procedure termed the “meta-challenge”:

a) Divide the theoretical space into realist and eliminativist theories, and then divide those into those accepting a solution to the meta-problem and those that do not.
b) Challenge eliminativists to provide a topic-neutral solution to our anti-eliminativist intuitions.
c) Challenge consciousness realists that accept a solution to the meta-problem to defend themselves from charges of unjustified belief.
d) Challenge consciousness realists rejecting the existence of a solution to the meta-problem to explain their choice (as it would likely entail that zombies are inconceivable and that machines cannot pass the Turing Test).

Applying this procedure to different theories of mind is outside the scope of this paper. What is important to the purpose of this paper is that the meta-challenge puts real pressure on current theories of mind, so much so that it causes Chalmers to be pessimistic about their prospects.

Solutions to the meta-problem must be realized by some brain process that we might call the “meta-process,” just like solutions to the hard problem by some “consciousness process” (Chalmers, 2018). If the two processes are identical, one is forced to embrace eliminativism (Frankish, 2019); if they are different, one must defend charges of unjustified belief. Chalmers’ own preferred solution is “realizationism,” by which consciousness is realized by meta-processes or where phenomenal consciousness is realized by access-consciousness. In the next section I will argue against realizationism and for what Chalmers terms “Meta-Correlationalism,” by which the meta-process and the consciousness process are separate but perfectly correlated. These theories are problematic when the correlations are brute, like in dual-aspect theories of consciousness. Chalmers’ own dual-aspect information theory of consciousness, by which information has a phenomenal aspect and a material aspect, at the same time suggests that the same information is simultaneously realized mentally and materially. The holographic theory that I pursue here is partly similar to this, and yet very different as now *the two aspects are correlated and necessarily so.*

Even if the categorical basis of microphysical properties were phenomenal, that would not explain how consciousness starts flipping electrons so as to use the brain to proclaim its existence. However, “meta-challenging” Russellian Monism (RM) shows that it fails to overcome charges of epiphenomenalism (as it “promises” to do) since we still have no idea how an intrinsic and non-relational categorical basis may act on the “molecules of the brain” to announce itself relationally. To make things worse, solving the notorious combination problem of type-F theories does not seem to help with the meta-challenge. Both ways out for realizationism considered by Chalmers are problematic. Interactionist Dualism (Crick and Koch, 1990) has insurmountable problems with causal closure (introducing new metaphysical and explanatory gaps), and the attribution of problematic “phenomenal powers” to the categorical base (Morch, 2020) not only defeats the purpose of Russellian monism but raises a novel problem. Were phenomenal powers to exist (say they could flip an electron’s spin), then one could design experiments showing that during conscious reports it is possible to establish the existence of extra-theoretic influence on the results of measurements performed on the physical substrate underlying such reports. I do not see this happening anytime soon.

In the next section I will present some new and surprising connections between quantum gravity and condensed matter theory and argue that they possess the resources to provide topic-neutral explanations to the aforementioned problem intuitions.

## Holographic Duality and Strange Metals

### Duality

In their *Introduction to Special Issue on Dualities*, Castellani and Rickles (2017) begin with “dueling concepts” in the Chinese cosmology and compare these to an analogous duality in science:

In Chinese cosmology, the various phenomena of the universe are then viewed as an interplay of these dueling concepts. Further, the two sides are inextricably entangled and interdependent, neither making complete sense without the other: there is no sense of one side of the dual pair being more fundamental or superior. So seems to be the case with dualities in science, with a similar entanglement holding together dueling concepts such as “hot/cold,” “big/small,” “high-energy/low-energy,” “finite/infinite,” “composite/elementary,” “localized/delocalized,” etc., though in such a way that an equivalence holds between them at some level— in general, one finds that some quantity and its reciprocal are involved in a duality mapping. There is, then, mystery in dualities, in making sense of how there can be equivalence given such apparently stark differences (differences labeled, in this special issue, as “striking” by De Haro and “shocking” by Huggett). Yet, within this mystery there lies a golden opportunity for philosophers.

Duality in mathematics and physics is not a theorem or a law of nature but a deep principle (Atiyah, 2007) that excels at “carving nature at the joints,” so to speak; in mathematics it has been used for hundreds of years and continuously adapted and evolved, undergoing successive generalizations that guided progress in geometry, algebra and analysis. In physics, duality entered the scene with Maxwell’s equations’ invariance to a duality transformation that exchanges the electric field E with the magnetic field B and B with –E when no sources are present, later with wave-particle duality in quantum mechanics, and finally with the Kramers-Wannier duality in 1941 that yielded a simple way to predict phase transitions in the 2D Ising model. Quantum field theory then presented us with new dualities, like the “Montonen Olive Duality” in 1977, while dualities became central to string theory – with S-Duality, T-Duality and especially the mysterious M-theory, in which all five different string theories were shown to be cross sections of the same currently unknown theoretical object-related to each other by duality transformation. The E–B and KW dualities are self-dual, in the sense that the duals in these theories are described by the same equations. However, most dual descriptions are not isomorphic and the duals that I will be interested in here, is the AdS–CFT correspondence (Maldacena, 1998), or “gauge-gravity duality,” relating a many-body strongly interacting quantum field theory on a d-dimensional surface to a gravitational theory in “the bulk” with d+1 dimensions. The theory is related to the holographic principle in string theory, stating that the information of the physical “bulk” in d+1 dimensions is inscribed on its d-dimensional boundary. This String–QFT duality is surprising; to quote Polchinski (2015):

Dualities between field theories, and dualities between string theories, are remarkable, taking QFT and string theory far beyond their perturbative descriptions. A duality between a field theory and a string theory might seem to be impossible, on several grounds. String theories require ten dimensions, whereas renormalizable field theories do not seem to exist in dimensions greater than four… String theories seem to contain many more degrees of freedom than QFT’s, from the infinite number of internal states of the string. And, string theories contain quantum gravity, with its many conceptual puzzles, while renormalizable QFT’s do not. Well, prepare to be amazed.

Duality in physics can be seen as a symmetrical transformation relating different *theories* of the same entity. Unlike ordinary global and local symmetries that leave physics invariant with respect to solutions to the same theory, under this duality the observable physics is invariant under exchange of theories. Duality is therefore a more radical symmetry that can relate different physical theories in different space dimensions, while maintaining a strict but highly counter-intuitive bijective mapping between fields, expectation values and other relevant physical variables.

The more we know about the structure of physics and math, the more central the role duality plays in acquiring this knowledge. However, the connections between the notion of duality and philosophy, especially philosophy of mind, are less well established (Castellani and Rickles, 2017):

Despite their ubiquity and importance in physics, mathematics, and logic, it is fair to say philosophers have yet to embrace dualities with the gusto they deserve. This is particularly unfortunate since dualities connect to a great many issues that philosophers have fully embraced. To name a few notable examples:
• Reduction, emergence, and fundamentality.
• Theoretical equivalence and synonymy.
• Underdetermination and empirical equivalence.
• Realism versus anti-realism.
• Unification in (and of) mathematics and physics.

To which one can add dual aspect theories (Vistarini, 2017), identity, and, which interests us here, the meta-problem of consciousness. One can also try to use duality as a meta-theoretic tool to capture the relationship between different theories of mind. For example, one might use the “relativity of fundamentality principle” (Castellani, 2016) by which what is fundamental on one side of the duality becomes composite on the other one.

The reason that the AdS-CFT correspondence is “holographic” is metaphorically represented by the holographs of 3D glasses; in the same way that the unintelligible interference patterns of the quantum fields on a two-dimensional surface are equivalent to, or dual to, an intelligible classical/phenomenological theory with an extra space dimension, the 2D glass hologram in linear optics, with its unintelligible printed interference patterns, is equivalent (in informational terms) to a 3D holograph made up of elements we may relate to. In a way, the 3D holograph is a geometric interpretation of a 2D field theory, serving, in that sense, a powerful parallelism for the AdS-CFT duality and the more general gauge-gravity duality. The difference is that, in the case of classical holography, the hologram with the use of a laser generates the holograph, which is not true in the case of more symmetric dualities like AdS-CFT.

### Physics With Resources to Explain the Structural Mismatch Problem and ∼P Problem Intuitions

If the most concrete thing we know –consciousness—is physical, akin to a novel exotic phase of matter, then the “physics of consciousness” must look very strange, perhaps one that will deconstruct matter, discover novel reflexive states, rely on subtler notions of identity and clarify the relationship between spacetime, information and entanglement. The most pressing problem in this respect is probably to relate such a putative physics of consciousness to the physics of spacetime and information.

One can argue that classical physics, including non-linear dynamics and electromagnetic theory, lack the resources to describe such states of matter, as do QM and QFT. I am well aware of QM (Stapp, 2007) and QFT theories of consciousness (Ricciardi and Umezawa, 2004; Freeman and Vitiello, 2006), and yet I believe that they lack the resources to solve not just the hard problem but also the meta-problem. After all, one of the biggest obstacles on the path to a “physics of consciousness” is relating it to space and time: we have no idea how to do that. Can information be realized in ways that transcend our ordinary conceptions of space and time? However, even if we consider background-independent physics, it is hard to see how some stringy version of quantum gravity can be used in a direct assault on the hard problem.

This is where the meta-problem comes into play. We can ask an “easier” question that is still relevant, i.e., “Can QM and QFT solve the meta-problem by providing topic-neutral explanations of our problem intuitions?” Chalmers (2018) suggests that they do not and as I argued in the prolog such S-facts are worth pursuing.

Is the physics of quantum gravity and the Planck scale up to the task and can it provide us with a solution to the meta-problem and tangible philosophical advantages?

The superstring revolution started in the 1960’s with the hope of reaching a theory of everything, thus explaining the origin of spacetime by unifying the standard model, super-symmetry, QFT and local gauge theories with gravity and Einstein’s equation. The theory had spectacular early success in areas like enumerative geometry, making substantial contributions to mathematics which won a string theorist like Ed Witten a Fields Medal. However, string theory was very frustrating i, because, despite its beauty and the deep connections it had with the foundations of mathematics and physics, the objects it predicted could not be verified experimentally. For example, experimental verification of the existence of mini black holes predicted by string theory would require a particle accelerator 100 times bigger than CERN. Any attempt to relate the physics of string theory and the AdS–CFT correspondence to the meta-problem and the hard problem must explain how Planck scale physics 20 orders of magnitude smaller than a proton may be relevant to the brain. After all, it is hard enough to establish the existence of non-trivial quantum effects in biological systems at room temperature. Even if Planck scale physics and quantum gravity did have the resources to solve the meta-problem, we would first need to show that this physics is relevant to macroscopic systems describable by Condensed Matter Theory (CMT), and then show that such peculiar CMT mechanisms are instantiated in the brain, preferably by processes that correlate with consciousness. In the last 20 years, the discovery of totally unexpected and even mysterious connections between CMT and quantum gravity has transformed both fields in a way that made them essential to each other. String theorists can finally perform laboratory experiments to verify their predictions and learn strange new facts about spacetime, black hole thermodynamics and the information paradox. Condensed matter theorists, in their turn, can use the sophisticated string theory mathematical machinery to understand highly entangled, strongly interacting novel states of matter that cannot be described by standard perturbative CMT. The possibility of a connection between such CMT states of matter and the brain is currently a useful thought experiment relevant to both the possibility of a physical solution to the meta-problem.

### Strange Metals

In 2007 Sachdev and his collaborators (Hartnoll, 2007) were trying to understand the properties of 2D high-temperature superconducting Copper-oxides named “Cuprates” (rusted copper) that displayed quantum critical behavior, namely scale invariance which made them describable by a conformal quantum field theory in two dimensions. When these materials were heated above their superconducting phase, they displayed unexpected transport properties (Nernst effect) and an electronic state of matter that can be described as “irreducibly many particle entangled compressible matter” (Zannen, 2018). These “Cuprates” were dubbed “strange metals,” because this electronic phase of matter exhibited conduction without quasi-particles and fast hydrodynamics typical of quark-gluon liquids. The use of standard perturbative QFT approaches proved intractable but they realized that a conformal QFT has a gravitational dual with an AdS4-CFT3 correspondence. Defying reason, they decided to borrow from the mathematical machinery of string theory and perform the same calculation in the dual gravitational space with an extra dimension containing a dyonic black hole (with charge and magnetism), itself a solution of the Einstein-Maxwell equations. To their surprise, the calculation explained the peculiar transport of heat and electricity typical of these “strange metals” and agreed with experiments. In the beginning they must have thought that this is a case of the same math coincidentally describing completely different phenomena and that the dual gravitational space was not real in any sense, but their findings drew more attention and scrutiny—bringing a flood of results that is impossible to review here. Let us just say that these results connected the “strange metal” state of such “Cuprates” to many electron states with Planckian dissipation and minimal viscosity, which is typical of quark/gluon plasmas and fast hydrodynamic configurations, maximally entangled states, “instant thermalization” and maximal entropy production that sets limits on physical computability (Lloyd, 2000). On the AdS side of the duality, strange metals became important as quantum computing devices, enabling string theorists to test string-based hypotheses about quantum gravity, black hole thermodynamics, the information paradox, the emergence of spacetime and more. As an example of the unreasonable success attributed to what Zaanen et al. (2012) terms the AdS-CMT correspondence (with Condensed Matter Physics):

*It is perhaps the greatest success of the AdS/CFT correspondence that Einstein gravity can be used as “generating functional” to determine hydrodynamical equations. The above is of course a particular basic example but at present this “method” is used with significant practical consequence, by re-assessing particularly complicated forms of hydrodynamics.*

Strange metals also provide a strange and novel form of computation, to quote Zannen (2018).

*We have come to the realization that holography is a mathematical machine that computes observable properties of stuff that is some kind of most extreme, “maximally” entangled form of this compressible quantum matter. Its observable properties do represent “physical” physics. However, this can be very different from anything that you learned in college.*

### Anti-de Sitter Space—Conformal Field Theory. Correspondence as Computation

Philosophy is written in this grand book — I mean the universe — which stands continually open to our gaze, but it cannot be understood unless one first learns to comprehend the language in which it is written. It is written in the language of mathematics, and its characters are triangles, circles, and other geometric figures, without which it is humanly impossible to understand a single word of it; without these, one is wandering about in a dark labyrinth (Einstein et al., 1935; Galileo, 1623).

As we said our search for the “mathematics of consciousness” did not appeal to novel mathematics and “first principles”; pretty much the opposite: first, we looked for physics that is rich enough to solve strategically chosen aspects of the meta-problem, and only then asked ourselves whether it has interesting computational or mathematical properties. I argued that the only physics that can solve these-“meta problems” finding room for a non-spatial mind in a spatial world is a background independent physics like the one we find in quantum gravity. It turns out that at this level information becomes more basic than space and time and that’s an advantage for a possible “physics of consciousness.” Any physical process computes the temporal evolution of its own states and can be viewed as an analog computer. Useful computing devices operate on “unmanageable” input that we cannot directly relate to, to produce, or distil, output that is relevant to us and that we can relate to.

Shortly after Maldacena’s discovery of the AdS-CFT correspondence, Gubser, Klebanov and Polyakov, followed by Witten, discovered the GKPW rule (or transformation), providing a precise mapping between the physics on both sides of the duality and a universal dictionary of sorts.

*“…the dictionary is also greatly counterintuitive and after all these years still seen as in many regards quite mysterious even by the professional holographists”* (Zaanen et al., 2012).

This is also where the interest of the string theorists resides: “the dream is that condensed matter experiment might be used as an analog quantum computer to test the weak conjecture under circumstances where one does not know how to proceed mathematically” (Zaanen et al., 2012). We see then that a rusted piece of copper may be viewed as performing “analog quantum computation.” This can be used to not only test the validity of the dictionary, but also to make novel predictions.

The AdS-CFT correspondence acts like a computational device that transforms unintelligible “hard” quantum information into “easy” classical information. Commenting on the physicists’ “discovery” of mirror symmetry that Atiyah (2007) attributes to duality between complex geometry (for example, Riemann surfaces and algebraic varieties over the complex numbers) and symplectic geometry (for example, real manifolds that generalize the phase space of classical Hamiltonian mechanics), he concludes:

So this marvelous theorem tells us that easy information on one side (periodic matrices, that can be calculated by classical means) is equivalent to difficult information on the other side (algebraic curves, for whose determinations there is very little information). In physics language, the easy information is what is called classical and the difficult one is what is called quantum. We are thus getting information of a quantum character on one side out of classical calculations on the other side.

Similarly, Zaanen et al. (2012) describes holographic duality as transforming an intractable strongly interacting QFT into a more phenomenological theory, based more on things that we know and can relate to:

Our interest is in the behavior of an infinite number of strongly interacting quantum degrees of freedom, and “holography” appears as a “generating functional” that is supposedly extremely powerful in revealing the principles controlling “deep emergence” physics, translating it into phenomenological theories of a Landauesque quality.

Concluding:

From the viewpoint of the mathematically inclined string theorist, this is where the relevancy of the present flirtation with condensed matter physics resides: the dream is that condensed matter experiment might be used as an analog quantum computer to test the weak conjecture under circumstances where one does not know how to proceed mathematically.

### Einstein-Rosen Bridge = Einstein Podolsky and Rosen

The strongest version of the AdS-CFT correspondence is probably advocated by Leonard Susskind (2017), adopting a literal interpretation of the ER = EPR principle that relates EPR, (Einstein et al., 1935) and quantum entanglement to the Einstein-Rosen bridge. On this account, entangled fields in a d-dimensional QFT serve as boundary conditions to a dual d+1 geometric space, with gravity and connected by wormholes. This is a case of metaphysical necessity and not a case of ordinary empirical equivalence, because entanglement on the surface cannot exist without the wormholes in the bulk and vice versa. The duals do not emerge from each other and are neither grounded in nor constituted by each other but share a more symmetric form of equivalence reminiscent of dual-aspect monism (Vistarini, 2017). For Susskind (2017), any entanglement is accompanied by a corresponding wormhole and he envisions: seeing quantum gravity in a lab equipped with quantum computers and expecting that these will become feasible sometime in the next decade or two.

He then considers a thought experiment including a spherical shell that operates as an elaborate quantum computer. It instantiates a conformal QFT and serves as a boundary to an equivalent “bulk” dual with an extra dimension. The dual extra dimensional space with gravity (“projected” by the boundary CFT) is a necessary consequence of the dual physics. This “bulk” dual space is different than lab-space, and Susskind speculates about the possibility of an observer that is simulated by, or “merges” with, the 2D quantum computer in the shell, thus also entering the dual space to interact back with us. This is why such strong holographic duality can explain the ∼P Problem intuition, as entering the shell in the lab does not grant access to the “bulk” space dual to the surface quantum computer; to gain access to the “bulk,” the technician must first be converted into a computer program that is inserted into the computer. For the same reason, if our (P)NCC were to consist of some complex conformally entangled CFT state, say with two-dimensional properties, it would be dual to a 3D “bulk” space that cannot be detected in the “lab-space” of the brain – unless you manage to somehow incorporate an external observer into the 2D “quantum computer.” This works just as well with a 3D CFT and a 4D AdS. Susskind’s take on entanglement suggests viewing such ‘private’ AdS spaces as precisely the kind of “Island Universes”” that Lewis argued against. Here consciousness is constituted like space (action of renormalization in CFT) and owes its robustness to a quantum error-correction code that may be different than the one instantiated by ordinary space.

### Holographic Duality and Quantum Error Correction Codes

Preskill (2012) defines “quantum information science” as exploration of the frontier of highly complex quantum states, the “entanglement frontier”. Such systems cannot be simulated by classical computation or given a tractable mathematical description (Feynman, 1982) and can only be simulated with a quantum computer consisting of entangled qbits. N qubits live in a 2*^N^*-dimensional Hilbert space and, if a gram of highly entangled matter contains Avogadro’s number (10^23^) of such qbits, it generates a fantastically big “frontier” with a (2^10^23^^) dimensional Hilbert space. Achieving such “quantum supremacy” demands an efficient unitary quantum error correction code and several such codes are currently being considered. Here the AdS-CFT correspondence and holographic duality keep providing novel dazzling insights into the workings of reality. The central mystery of the AdS-CFT correspondence is the “emergence” of gravity and “projection” of the additional spatial dimension (or time dimension in dS-CFT) that dualizes into “renormalization flow” and coarse graining:

It is called “holographic” since the gravitational side has one extra dimension: this “radial direction” connects the boundary to the deep interior [of the de-Sitter space] and has the identification as the scaling direction in the field theory. The claim is that AdS/CFT geometrizes the renormalization group and upon descending deeper in AdS one “sees” the physics at longer times and distances. The deep interior codes for the macroscopic scale (“IR”) (Zannen, 2018).

Here Preskill and others (Patawski et al., 2015) show that the holographic correspondence establishes an equivalence between quantum error correction codes operating on the boundary QFT and the “robustness” of spacetime. Quantum error correction codes are based on entanglement and protect the information in individual qbits from noise by embedding this information in the entanglement patterns of multiple qbits. Spacetime itself is seen as constituted by entangled qbits of sorts and holographic duality strongly suggests that it owes its stability to a very efficient error correction code: “If such a code exists it must be very special and DARPA, taking notice, is funding efforts to discover such codes with the hope of producing efficient quantum error correcting codes to achieve ‘quantum supremacy”’ (Wolchover, 2019). The surprising connection between quantum computation, error correction codes and the constitution of spacetime, made clear by the AdS-CFT duality, is just the latest demonstration of duality as a gift that keeps on giving. If there is a “physics of consciousness,” then it may be related to the physics of time.

## Discussion

The Strange Metal Theory (SMT) is a consciousness realist theory that handles the meta-challenge by accepting a separate spatiotemporal unfolding of the “radically different” consciousness process and meta-process—while showing that they realize the same information. I believe that what we know and say about consciousness has something to do with the way consciousness *really is*, and showing that we would probably make conscious and problem reports even if we were not conscious threatens this belief and the very foundations of our self-knowledge. However, SMT relies on two parallel processes realizing the same information. Think of a hologram varying continuously in time, creating a 2D hologramic movie of sorts, and consider its dual 3D holographic counterpart: in SMT these movies cannot act on each other in any way and a frame in one movie can only be influenced by other frames in the same movie. Entities in one space may map in a complex and non-local manner unto dual entities in the dual space that may be very different.

While it’s true that the action of vocal cords generating “conscious reports” are not caused by consciousness but by very different non-conscious 2+1 or 3+1-dimensional processes describable by CFT, in the corresponding 3+1- or 4+1-dimensional dual AdS “phenomenal” space (here a topic-neutral space that can be given a more classical/geometrical interpretation), the conscious reports are caused by consciousness.

If parts of a minimal (P)NCC were shown to display “strange metal” dynamics, that would immediately suggest a solution to the SMP and ∼P Problem intuitions. The SMP can be explained by the way that holographic duality manages to conceal the necessary connections between the duals (fact used by Uri Geller in Sec. “Ineffable and Manageable Anti-physicalist Intuitions”) and the ∼P Problem (also see Sec. “Ineffable and Manageable Anti-physicalist Intuitions”) can be explained by the strange ontological nature of such dual spaces. Remember Susskind’s ER = EPR sphere in which the entangled CFT on the shell in the lab-space demands the existence of a “bulk” physics with an extra spatial dimension and gravity that is just as real but not identical to the lab-space associated with the interior of the shell. When measuring devices that live inside the 2D space perform measurements in this space, dual corresponding measurements must occur in the “bulk”; in order to access that strange “bulk” space you have to live in the 2D shell space, and this is why Susskind’s lab technician must merge with the 2D “quantum computer” shell that simulates her in two dimensions (must be uploaded into the 2-D quantum computer) to access the 3D “bulk.” If the NCC is governed by holographic duality and I will shortly consider a similar 2+1-D NCC governed by CFT with a 3+1-D dual, the only way to access the 3D dual space is “inhabiting” the 2D space. In any event the dual 3D space is not identical to the laboratory space in the way the interior of our skull is. That would also explain why our phenomenal space can only be accessed by us, how two completely different things can be identical and the intuition that consciousness cannot be identical to anything in the brain. Susskind’s radical interpretation of entanglement and the AdS-CFT correspondence is not shared by everyone For example Verlinde (2011) holds that the duals are not completely symmetric. Granting this, the extra space dimension on the AdS side of the duality emerges from the boundary CFT in the same way that thermodynamic variables emerge from microscopic physics. However, Susskind’s position is sound, and radical enough to provide a physical explanation to our ∼P Problem—which is otherwise very difficult to explain.

Tononi (2008) claims that any system with non zero Phi has inner properties that are only accessible to the system itself but the claim is brute, put in by hand so to speak, and does not result from the physics: likewise Varela’s autopoietic approach to biological organisms fails to establish any extra-theoretic inner goings on above and beyond those described by ordinary molecular biology (Dennett, 2011). However, if Suskind is right and the only way a “technician” can enter the “geometric” bulk space is by being “uploaded” unto the 2-Di quantum computer then such AdS spaces can harbor representationally rich “subjective physical facts.” Discovering that our minimal NCC harbors such facts can explain aspects of the privacy of our mental experiences and be philosophically significant. It seems though that we still face a hard problem of consciousness. The dual classical, geometric space is still physical, and we have to explain how consciousness can be identical to something physical whether in that space or in any space. The deep idea here is that scaling with a proper renormalization theory in the CFT creates space in the AdS and if the conscious field is a sort of space that does reside in the “lab-space” then this is the way to construct it. The theory also deconstructs matter and explore the prospects of a mathematical transform that actually solves the SMP by generating structural aspects of the original environmental input (we do not need different individuals to harbor identical phenomenology, rather only a one to one mapping between them that maps unto a common environment). Discovering such a transform would mean that we have managed to identify the isomeric physical correlates of representational content, thus putting us in a better position to understand the physics of phenomenal character. One of the advantages of a topic-neutral physical solution of the meta-problem over other structural topic-neutral solutions to the meta-problem is based on a parsimony argument. Both representational content and phenomenal character are special and novel phenomena. It is unlikely that two highly correlated strange and novel phenomena be generated by two unrelated novel and strange mechanisms. That is, if you discover the physics of representational content, it will probably contain important clues that can help with the phenomenal character problem. If, on the other hand, we find solutions to the meta-problem that are based on standard computational approaches, it is hard to see how the same class of algorithms that would shed light on the SMP and representational content in general may put you in a better position to solve the phenomenal character problem. After all there is a difference between the map and the territory. Also, the physical solution to the ∼P problem intuition based on holographic duality cannot be simulated but only *generated* by the right quantum computer.

What about zombies? Suppose we find out that aspects of a minimal (P)NCC instantiate holographic duality *à la* Susskind: would that make zombies inconceivable? Suppose that we also discover that the same mathematical transform explaining the ∼P Problem intuition (by providing a private AdS space) also solves aspects of the SMP (by showing that the transform preserves elements of the environmental structure), would that make zombies inconceivable? Seems like we can still have holographic “strange-metal” zombies or “AdS zombies” but these are considerably less likely. One reviewer insisted, and rightfully so, that showing that zombies are “highly unlikely” is not enough to undermine physicalism. Here I think that mature sciences and epistemologies determine their scope and limitations from the inside, so to speak, Heisenberg’s Uncertainty and Gödel’s Undecidability are a case in point. Perhaps brain science needs to mature similarly by collaborating with condensed matter physics to establish the existence of (P)NCC systems that contain rich representations that cannot be accessed by ordinary physical measuring devices and necessarily so. I believe that while improving our “epistemic condition” such a scenario still leaves open questions about phenomenal character that makes “holographic zombies” conceivable. What I am suggesting is that discovering that the (P)NCC is one of those rare systems in which information can be realized subjectively can provide us with clues about phenomenal character and the nature of this exotic state of matter. The need to appeal to a background independent physics in order to “place” non-spatial mind in a spatial world suggests a neutral-monistic q-bit based token-physicalism in which the ultimates are not multiply realizable. Such possibly protophenomenal ultimates/qbits can be realized either objectively/scientifically or subjectively/manifestly *and for physical reasons!*. In Awret (2019) I consider the AdS duals of self-referential processes on the CFT surface such asLloyd (2000) gates which emulate closed time-like curves. So yes, holographic zombies cannot be ruled out but discovering that a minimal (P)NCC instantiates AdS correspondence in a way that solves both the ∼P and SMP problems should point us in the right direction. Parsimony suggests that the same novel mechanism responsible for the confinement of these subjectively realized q-bits is also responsible for their intimate and “luminous” self-access which should make us optimistic. If the same physical mechanism would explain the ∼P and SMP problems while also causing ultimates in the brain a novel kind of self-access AdS zombies would still be conceivable but these are not going to threaten physicalism in the same way if physics discovers private physical spaces associated with the (P)NCC and commensurate with its representational content that are inaccessible to it (external measuring devices). For the conceivability argument to work knowing the *totality* of physical facts implies knowledge of psychological facts but if some of the relevant facts are missing the argument fails. Speculating about the nature of inaccessible facts cannot be used to undermine physicalism.

We began with Castellani and Rickles (2017), tracing the origin of duality to the Yin Yang principle in ancient Chinese cosmology, and I will end with a related possibility. The Claustrum is a good NCC candidate (Crick and Koch, 2005; Torgerson et al., 2015; Reardon, 2017). Suppose that we discover that the Claustrum contains massively entangled electrons describable by a 2D CFT with a dual 3D AdS space in which our phenomenology unfolds. At some point we discover that the information in our 3D universe is actually inscribed on the former surface; then we begin to understand the Claustrum as a surface existing in our “imaginary” 3D space that is actually dual to that space. So, the 3D space harbors the 2D surface that generates it, a bit similarly to a Klein bottle (Brown, 2007). This kind of convoluted topology is reminiscent of the Yin Yang cosmology where each dual harbors a bit of the other dual.

## Data Availability Statement

The original contributions presented in the study are included in the article/supplementary material, further inquiries can be directed to the corresponding author.

## Author Contributions

The author confirms being the sole contributor of this work and has approved it for publication.

## Conflict of Interest

The author declares that the research was conducted in the absence of any commercial or financial relationships that could be construed as a potential conflict of interest.

## Publisher’s Note

All claims expressed in this article are solely those of the authors and do not necessarily represent those of their affiliated organizations, or those of the publisher, the editors and the reviewers. Any product that may be evaluated in this article, or claim that may be made by its manufacturer, is not guaranteed or endorsed by the publisher.

## Footnotes

1 To a first order approximation I will assume that reality exists, that it is structural and that our perceptions of the world are veridical to some extent (say, because of evolutionary pressure). While a bee and a primate may have different phenomenal character to a consciousness realist “phenomenal character” must refer to some minimal set of properties.

2 I believe that “biting the bullet” on strong emergentism should be an act of the last resort. This is certainly true for giving up on necessary connections between two structural domains that are exquisitely correlated. Also in Susskind’s cell the hardware of the quantum computer is not multiply realizable in the sense that a computer that simulates complex entanglement without itself being a similarly entangled quantum computer will fail to produce the bulk dual. Also the evolution of the relevant states of such a machine is only tractable to the bulk (Preskill). Similarly to Joseph Levine (2019). I loosen-up the metaphysical definition of identity to preserve necessity.

3
http://www.talete.mi.it

4 Sartre coherently linked the “being-for-itself” intrinsically to nothingness, as it was a hole in being, so to say. Also see Chalmers (2018) on attempts to solve the meta problem that appeal to “primitive property attribution.”
